# The relationship between retirement, social isolation and loneliness: A longitudinal analysis using the English Longitudinal Study of Ageing

**DOI:** 10.1186/s12889-025-24472-8

**Published:** 2025-11-03

**Authors:** Laura Kenny, Niamh Doherty, Ann Sinéad Doherty, Brian Lawlor, Roger O’Sullivan, Gerard Leavey

**Affiliations:** 1 The Bamford Centre for Mental Health & Wellbeing, School of Psychology, Ulster University, Belfast, UK; 2 School of Psychology, Arden University, London, UK; 3 Department of General Practice, School of Medicine, University College Cork, Cork, Ireland; 4 The Global Brain Health Institute, Trinity College Dublin, Dublin, Ireland; 5 The Institute of Public Health, Dublin, Ireland

**Keywords:** Loneliness, Social isolation, Retirement, Wellbeing, Ageing

## Abstract

**Background:**

Retirement is a major transition in aging, with changes in routine, identity, finances, and social connections, and subsequently mental and physical health. It is possible that the transition through retirement period may increase the risk of loneliness and social isolation. However, the relationship between retirement and loneliness is unclear. We aimed to examine the association between retirement and loneliness and social isolation using a representative sample of older adults.

**Methods:**

We used survey data from the English Longitudinal Study of Ageing (ELSA), a representative sample of adults over 50 years of age living in private households in England. We analysed waves 4–8 covering the years 2008 to 2017. Loneliness was measured using the UCLA 3-item scale. Social isolation was measured using information on social connections based on previous methods used in ELSA. Binary logistic regression models were used to analyse the effect of retirement on loneliness and social isolation.

**Results:**

From a total of 3,758 participants, 766 retired between wave 4 and wave 5. Retirement had no effect on short-term loneliness (adjusted odds ratio (aOR): 0.96, 95% CI: 0.77–1.21), but did reduce odds of social isolation (aOR: 0.76, 95% CI: 0.58–0.98) for newly retired individuals when compared to those still working. Further, no association was observed in long-term analysis for either loneliness or social isolation.

**Conclusions:**

Our findings suggest there is no immediate or long-term association between retirement and loneliness, but there may be a reduction in social isolation in the short-term. Understanding the role retirement plays in the complex relationship between social connection, loneliness and isolation can be used to inform strategies and policies to improve wellbeing in older age.

**Supplementary Information:**

The online version contains supplementary material available at 10.1186/s12889-025-24472-8.

## Introduction

Loneliness is the feeling resulting from the subjective discrepancy between one’s actual and desired social and emotional relationships, closely related but distinct from social isolation referring to the objective lack of social connections and relationships [1]. Loneliness and social isolation have been associated with an increased risk of several mental and physical conditions, including depression [2]dementia [3]cardiovascular disease [4]in addition to all-cause mortality [5]. It has been suggested that these associations are partly mediated through the effect of health behaviours, such as smoking, physical activity and sleep, and stress pathways [6]. Further, loneliness in older adults has been associated with increased primary care visits [7, 8] and frailty [9]highlighting the impact that loneliness has on wider healthcare use. As such, loneliness has become a notable public health concern in recent years with support from the World Health Organisation (WHO) Commission on Social Connection launched in 2023 [10].

In the UK, 22% of adults report being lonely at least some of the time according to the 2021 Health Survey for England including 5,880 responses [11]. People in the most deprived areas reported higher levels of loneliness compared to those in the least deprived areas (33% vs. 10% lonely at least some of the time, respectively) [11]highlighting not only the potential impact of loneliness on individual health but also in shaping health inequalities. Levels of loneliness broadly declined with age from 26% of 16–34 years olds reporting loneliness at least some of the time to 16%−17% in the 65–74 and over 75 age groups [11].

Retirement has broadly been linked with beneficial effects on mental health [12]with the findings regarding physical health more contradictory [12–14]. However, the relationship between retirement and loneliness remains unclear. While the transition from working to retirement may reduce social contacts with colleagues [15]potentially leading to loneliness, some evidence suggests social connections are maintained and/or improved after retirement [16]. In addition, the quality and frequency of social connections in retirement may improve due to the additional time to engage in hobbies, and interests and to connect with friends and loved ones and may in turn lead to reduced loneliness [15]. This is supported by findings that show the majority (56%) of workers close to retirement look forward to retirement, however a large portion (32%) are worried about missing their social connections and 17% were worried about being lonely [17].

Further, the circumstances of retirement can alter the effect of retirement on loneliness, with previous studies indicating that involuntary retirement can lead to increased loneliness but receiving social support, especially from family, can mitigate this effect [18]. Therefore, those with stronger social support may have less stress during retirement than those with weaker support networks, suggesting that social isolation can amplify the negative impact of retirement on loneliness. However, little attention has been given to the interplay of these factors in early retirement. In particular, individuals experiencing social isolation may be more prone to loneliness following retirement. Therefore, understanding how social isolation influences loneliness among new retirees is important as this group may be particularly vulnerable during this period of change.

Analysis of the Survey of Health, Aging, and Retirement in Europe which included 13 European countries and Israel, found no short-term effect of retirement on loneliness but a reduction in loneliness in the long-term [19]. On the other hand, a longitudinal comparative study found differences in the effect of retirement in different countries, with Australia and the US experiencing higher levels of loneliness post-retirement and Chinese retirees experiencing less loneliness [18]. This may indicate the importance of the cultural context when examining the relationship between retirement and loneliness [18]with communitarian societies less impacted than individualistic ones.

Given the significant adverse effects of loneliness further understanding of the risk of loneliness for those entering retirement is essential to inform future interventions and reduce the burden of loneliness in older adults in the UK. We aimed to utilise longitudinal data from England to analyse whether retirement is associated with increased loneliness and whether this relationship is moderated by levels of social isolation in a UK population.

## Methods

### Sample

We used data from the English Longitudinal Study of Ageing (ELSA), a nationally representative study of adults aged 50 years and older residing in private households in England [20]. Coordinated by the National Centre for Social Research, the original sample was drawn from households that had previously responded to the Health Survey for England (HSE) between 1998 and 2001. Data collection for wave 1 began in March 2002 with subsequent waves collected every 2 years. Respondents participated in a face-to-face interview (a computer-assisted personal interview, followed by a self-completion questionnaire). Replenishment occurred at waves 3, 4, 6, 7, 8, 9, and 10 to maintain the size and representativeness of the panel. Ethical approval was granted by the London Multi-Centre Research Ethics Committee for waves 1 to 3, from the National Hospital for Neurology and Neurosurgery & Institute of Neurology Joint Research Ethics Committee for wave 5 and all subsequent waves were approved by the South Central – Berkshire Research Ethics Committee. The current study used data from 3,758 participants between wave 4 and wave 8, as wave 4 onwards included all required information on retirement. Participants were selected if they answered both wave 4 and 5 (defined as the baseline period) and had information on loneliness and retirement status. For longitudinal analysis they were additionally required to have answered at least one subsequent wave.

### Variables

### Loneliness

We used the shortened 3-item UCLA Loneliness Scale (UCLA-3) [21]. The scale asks respondents to indicate how often they felt ‘left out’, ‘isolated from others’, or lacked in ‘companionship’ using a 3-point Likert scale. Response options were ‘hardly ever/never’, ‘some of the time’, and ‘often’ which could result in a total score ranging from 3 to 9 with higher scores indicating greater loneliness. In line with previous analyses [22, 23]those scoring 6 and above were considered lonely.

### Social isolation

Social isolation was computed using a five-item index, following guidelines used in previous studies [9, 24]where one point was assigned if the participant reported having less than monthly contact (including telephone, written/email/text messaging or face-to-face contact), with friends, their children, and other family members respectively. In addition, one point was assigned if the respondent reported they lived alone and were not involved in any social clubs or organisations. Scores could range from 0 to 5 and were dichotomised at ⩾2 versus < 2 with higher scores indicating high social isolation.

### Retirement status

To capture those who recently transitioned into retirement the participants who reported a change in retirement status between waves 4 and 5 were identified. Specifically, individuals were classified as ‘newly retired’ if they reported being employed, self-employed, unemployed, permanently sick or disabled, or looking after home or family as their current situation in wave 4, and subsequently reported being retired in wave 5. Individuals who may have been temporarily away from work or outside formal employment for other reasons were still included in the analysis.

### Covariates

Models were adjusted for demographic variables including age, gender, marital status, education, wealth, self-reported health, and ethnicity when modelling both social isolation and loneliness. Participation in religious groups or organisations was additionally included as a confounder for models examining loneliness. These covariates were selected based on their established relationships with social well-being and potential to confound the relationship between retirement, loneliness, and social isolation. These variables were all measured at baseline (wave 5) and included in the regression models and moderation analysis.

### Analysis

We used descriptive statistics for the study population characteristics and the primary outcomes at baseline (wave 5). Binary logistic regression models were used to examine whether retirement status, determined between wave 4 and 5, would explain loneliness in subsequent waves. We analysed the short term effect with loneliness captured at wave 5 and long term effect with loneliness captured at the latest wave between waves 6–8. This analysis was then replicated to further examine retirement status and social isolation in future waves. To investigate the role of social isolation we ran a moderation analysis to measure the degree to which the relationship between retirement and loneliness was moderated by social isolation. Participants missing information on retirement status or any of the loneliness measures were excluded from the sample. For social isolation models participants missing information on any component of social isolation were excluded. Otherwise, missing data was handled using missing category for relevant covariates.

We ran sensitivity analyses on the robustness of our results. First, we created a new binary variable to indicate the change in loneliness between wave 4 (pre-retirement) and wave 5 (for short-term effect) and wave 8 (long-term effect), to investigate the change in loneliness after retirement. The new variable was coded as 0 indicating no change in or a lower loneliness score and 1 indicating an increased loneliness score. Second, we removed individuals who retired between waves 5 and 8 that were in the control group to ensure the long-term estimates were not driven by these individuals. Third, we removed individuals who retired prior to reaching the pension age as these individuals are likely to have different circumstances than the general population. Finally, we excluded those who were categorised as lonely at wave 4 (for short-term analysis) and wave 5 (for long-term analysis) to investigate whether retirement has an effect on the onset of loneliness. All analyses were done using STATA (version 18).

## Results

Following exclusions of those already retired, proxy interviews, and missing data of loneliness or retirement status, the study sample for short-term analysis consisted of 3,758 participants who answered waves 4 and 5, of which 56% were female (Table 1). For long-term analysis, participants were further required to have completed at least one wave between waves 6 and 8, resulting in a sample of 3,433 participants, with 706 categorised as newly retired. At baseline (wave 5), participants’ mean age was 58.4 years (6.9 SD) and 81% married or cohabitating (Table 1). Almost a fifth of participants were classified as lonely (UCLA-3 score 6 or above) at baseline. Newly retired were more likely to be single, had lower education levels, and reported poorer health on average when compared to unretired participants (Table 1). Overall, the prevalence of loneliness was 19.5% which remained the same in the working and newly retired population. The number of people reporting social isolation was slightly higher in the non-retired group compared to the newly retired population (17.8% vs. 16.2%, respectively).

**Table 1:** Baseline characteristics at wave 5

	Whole Sample *n* (%)	Not Retired *n* (%)	Newly Retired *n* (%)
Total Number	3,758	2,992	766
Age, mean (SD)	58.4 (6.9)	57.0 (6.0)	63.6 (6.8)
Female	2,117 (56.3)	1,662 (55.6)	455 (59.4)
Marital Status			
Married/cohabit	3,060 (81.4)	2,465 (82.4)	595 (77.7)
Neither	698 (18.6)	527 (17.6)	171 (22.3)
Education			
Higher	1,553 (41.3)	1,270 (42.5)	283 (37.0)
Intermediate	1,573 (41.8)	1,245 (41.7)	328 (42.9)
No Qualification	627 (16.7)	473 (15.8)	154 (20.1)
Self-reported health			
Excellent	626 (16.7)	525 (17.6)	101 (13.2)
Very good	1,302 (34.6)	1,049 (35.1)	253 (33.0)
Good	1,092 (29.1)	865 (28.9)	227 (29.6)
Fair	529 (14.1)	390 (13.0)	139 (18.2)
Poor	207 (5.5)	162 (5.4)	45 (5.9)
Physical Activity			
Low/sedentary	760 (20.2)	562 (19.3)	198 (26.0)
Moderate	1,990 (53.0)	1,585 (54.4)	405 (53.2)
High	928 (24.7)	769 (26.4)	159 (20.9)
Financial wealth quintile			
1	852 (22.7)	717 (24.4)	135 (17.9)
2	694 (18.5)	572 (19.5)	122 (16.2)
3	704 (18.7)	563 (19.2)	141 (18.7)
4	774 (20.6)	596 (20.3)	178 (23.6)
5	666 (17.7)	489 (16.7)	177 (23.5)
Part of a religious group			
No	3,035 (80.8)	2,455 (82.1)	580 (75.7)
Yes	586 (15.6)	436 (14.6)	150 (19.6)
Missing	137 (3.6)	101 (3.4)	36 (4.7)
Loneliness (UCLA-3) category			
Not lonely (3–5)	3,025 (80.5)	2,409 (80.5)	616 (80.4)
Lonely (6 or more)	733 (19.5)	583 (19.5)	150 (19.6)
Social Isolation category			
No (0–1)	2,979 (79.2)	2,368 (79.1)	611 (79.8)
Yes (2 or more)	642 (17.1)	523 (17.5)	119 (15.5)
Missing	137 (3.6)	101 (3.4)	36 (4.7)

Note. Quintiles of individual wealth was derived from net financial wealth (gross financial wealth removing financial debts)

Table 2 shows the odds ratios (OR) for the relationship between retirement status and subsequent loneliness and social isolation. New retirees were no more likely to report loneliness than the non-retired in the short- or long-term analysis (adjusted OR (aOR): 0.96, 95% CI: 0.77–1.21 and aOR: 0.95, 95% CI: 0.75–1.20, respectively). In the short-term analysis, newly retired participants were less likely to report social isolation compared to the non-retired (aOR: 0.76, 95% CI: 0.58–0.98). However, this effect disappeared in the long-term analysis (aOR: 0.84, 95% CI: 0.62–1.14).

**Table 2:** Odds ratio for the relationship between retirement and loneliness or social isolation

	Loneliness		Social Isolation	
	Unadjusted OR (95% CI)	Adjusted OR* (95% CI)	Unadjusted OR (95% CI)	Adjusted OR* (95% CI)
**Short-term (wave 4–5)**				
Not retired	1.00 (ref)	1.00 (ref)	1.00 (ref)	1.00 (ref)
Newly retired	0.95 (0.77–1.18)	0.96 (0.77–1.21)	0.81 (0.64–1.01)	0.76 (0.58–0.98)
**Long-term (wave 5–8)**				
Not retired	1.00 (ref)	1.00 (ref)	1.00 (ref)	1.00 (ref)
Newly retired	1.05 (0.85–1.29)	0.95 (0.75–1.20)	1.05 (0.80–1.38)	0.84 (0.62–1.14)

*Adjusted for: age, sex, education, marital status, self-reported health, physical activity, wealth, religious

Social isolation at baseline (wave 5) was associated with increased odds of loneliness among new retirees in both the short- and long-term, but this was attenuated when adjusting for covariates (aOR: 1.19, 95% CI: 0.71-2.00 and aOR: 1.01, 95% CI: 0.57–1.81, respectively; Table 3). We examined whether social isolation at baseline moderated the effect of retirement on loneliness (Table 4). The analysis did not indicate a moderating effect of social isolation on the relationship between retirement and loneliness for short-or long-term analysis ($\:\beta\:$: 0.2123 SE: 0.27, and $\:\beta\:$: −0.0105 SE: 0.30, respectively; Table 4).

**Table 3:** Odds ratio for the relationship between social isolation and loneliness, by retirement status

	Unadjusted OR (95% CI)	Adjusted OR* (95% CI)
**Short-term (wave 4–5)**		
Not retired		
Not socially isolated	1.00 (ref)	1.00 (ref)
Socially isolated	2.07 (1.63–2.63)	1.05 (0.78–1.41)
Newly retired		
Not socially isolated	1.00 (ref)	1.00 (ref)
Socially isolated	2.34 (1.49–3.68)	1.19 (0.71-2.00)
**Long-term (wave 5–8)**		
Not retired		
Not socially isolated	1.00 (ref)	1.00 (ref)
Socially isolated	1.85 (1.43–2.41)	0.98 (0.72–1.35)
Newly retired		
Not socially isolated	1.00 (ref)	1.00 (ref)
Socially isolated	1.75 (1.06–2.89)	1.01 (0.57–1.81)

*Adjusted for: age, sex, education, marital status, self-reported health, physical activity, wealth

**Table 4:** The moderating effect of social isolation on the relationship between retirement status and loneliness

	Coefficient (Standard Error)	Lower CI	Upper CI	*P*-value
**Short-term (wave 4–5)**				
Not retired	-			
Newly retired	0.2123 (0.27)	−0.3177	0.7424	0.432
**Long-term (wave 5–8)**				
Not retired	-			
Newly retired	−0.0105 (0.30)	−0.6059	0.5848	0.972

*Adjusted for age, sex, education, marital status, self-reported health, physical activity, and wealth

In sensitivity analysis, no significant changes were observed by using the alternative definition of loneliness, excluding participants who after wave 5, excluding those who retired prior to pension age, or excluding lonely at baseline (supplementary Tables 1–3 respectively, additional file 1). In addition, no change was observed in long-term analysis conducted separately for each wave (supplementary Table 5, additional file 1). Excluding marital status from the models for social isolation slightly reduced the aOR in both short and long-term analysis (supplementary Table 6, additional file 1).

## Discussion

This study examined the association between retirement and loneliness using longitudinal data from England. Our results suggest that there is no significant immediate or lasting association between retirement and loneliness. We found a reduction in the odds of social isolation among new retirees, however this effect did not translate to reduced loneliness in our analysis and appears to diminish over time. While retirement could potentially precipitate loneliness and social isolation for older adults [15]contrary to our results, it is possible that retirement creates opportunities for some people to strengthen connections with friends and family, at least in the early years of retirement; an effect that tapers off in later years possibly due to ill-health and disability. However, given the low number of individuals classed as newly retired it may be that we were unable to detect a relationship in long-term analysis. In addition, we did not find a moderating effect of social isolation on the association between retirement and loneliness. The findings were largely unchanged in our sensitivity analysis.

Previous studies on the effect of retirement on loneliness are mixed. A large study using panel data from the Survey of Health, Aging, and Retirement in Europe showed that retirement did not have a short-term impact on loneliness, supporting our findings, but demonstrated lower loneliness in the long-term when compared to the non-retired population [19]. In addition, those with higher levels of education showed a long-term decrease in feeling socially isolated and were more likely to participate in more activities [19]. It is possible that following retirement well-being, including loneliness level, is maintained or even improved [12, 25–27] especially in those with high education [19]. This highlights the social, physical, and financial inequalities that drive the discrepancies in the retirement experience by an individuals’ socioeconomic status. On the other hand, a study comparing the effect of retirement on loneliness in the US, Australia, and China found a differential effect by country, likely linked to contextual and cultural differences [18]. Findings indicated that retirees had higher levels of loneliness than non-retirees in the US, contradictory to our results, but in China the opposite was true [18]. In addition, those who retired voluntarily recorded lower levels of loneliness than those who involuntarily retired [18]. As age and retirement are so closely related, there are considerable challenges in isolating the effects of retirement on loneliness in addition to the individual and society level contextual factors that contribute to feelings of loneliness in this population.

Overall, our findings do not support the general negative perceptions of retirement that have previously been emphasised with respect to loneliness or social isolation [28]. Retirement is an important life transition which may offer new opportunities to connect and flourish in later life. Our findings support the potential for utilising this period to promote mental health and wellbeing in older adults through improving social connection. Interventions should consider promoting social engagement during and after retirement, potentially reducing the risk of prolonged loneliness in later life particularly for disadvantaged groups [19]. In addition, wider neighbourhood factors may be important pathways to improve social cohesion and loneliness in older adults, with particular attention to the role of neighbourhood safety [29].

### Strengths and limitations

This study used a large dataset representative of older adults (over 50) in England and used four waves of data to analyse the longitudinal association between retirement and loneliness. However, this study has some limitations. First, we did not examine how occupation or retirement type influenced post-retirement loneliness. Second, the relatively small numbers of newly retired participants limited statistical power and restricted subgroup analyses. In addition, residual confounding may be present, as geographic and wider economic factors were not included and time-varying covariates were not accounted for. This may reduce the specificity of our findings in specific contexts and could bias findings. Further, while marital status is a key factor in loneliness this overlaps with the social isolation measure used. We addressed this in sensitivity analysis excluding marital status. Third, it is possible that the maximum follow-up was not long enough to capture a change in loneliness and loss of participants may have biased findings, especially if individuals experiencing loneliness were less likely to remain in the study. This could potentially lead to an underestimation of loneliness in later waves. However, our sensitivity analysis separated by follow-up wave found similar findings to the main results. Fourth, it was not possible to determine the exact timing of retirement between waves. Thus, we were only able to observe that individuals become lonely within waves where retirement occurs. In addition, any participants missing loneliness or social isolation measures were excluded from the sample which may have introduced bias if they were not missing at random. Fifth, loneliness and social isolation were measured using binary variables which may have limited the insight provided by the variation in scores. Finally, the possibility of reverse causality cannot be ruled out, wherein loneliness can impact the decision to retire as well as result from it. Future research should explore more complex study design and methods that can account for individual differences and contextual factors not captured in this study, such as place of residence, personality, or finances.

## Conclusion

These findings contribute to the growing literature on mental health and retirement. This study did not find an association between retirement and loneliness in those who were newly retired compared to those who did not retire. However, our results suggest retirement may be associated with a small reduction in social isolation immediately following retirement. Retirement is an important milestone in older adults lives’ and provides the opportunity to interact with hobbies, leisure and social activities. For policymakers, leveraging this time to further improve loneliness, social isolation, and broader wellbeing will be of interest in identifying strategies to reduce the healthcare burden in this population. In addition, the older working population may also benefit from targeted interventions that address loneliness and social isolation prior to retirement. The complexity of retirement as a life transition in older age requires further research to identify the social and emotional factors, including loneliness, that contribute to well-being in older age.

## Supplementary Information

Below is the link to the electronic supplementary material.

Supplementary Material 1

## Data Availability

ELSA data is available from the UK Data Archive and are widely available.

## Abbreviations

CI: Confidence Interval
ELSA: English Longitudinal Study of Ageing
HSE: Health Survey England
OR: Odds Ratio
UK: United Kingdom
