# Supplementary material for: The relationship between retirement, social isolation and loneliness: A longitudinal analysis using the English Longitudinal Study of Ageing
Source: BMC Public Health. 2025 Nov 3;25:3733. doi: 10.1186/s12889-025-24472-8 (PMC12581352; doi:10.1186/s12889-025-24472-8)
Supplement: Supplementary file 1 — Supplementary Material 1 [file 12889_2025_24472_MOESM1_ESM.docx]

**Supplementary Tables**

Supplementary Table 1. Sensitivity analysis for the relationship between retirement status and loneliness, with alternative loneliness definition categorised as a change in loneliness score from wave 4-5 (short-term) and wave 4-8 (long-term)

Supplementary Table 2. Sensitivity analysis for the relationship between retirement status and loneliness, excluding those who retired between wave 5 and wave 8

Supplementary Table 3. Sensitivity analysis for the relationship between retirement status and loneliness, excluding those who retired prior to reaching the pension age

Supplementary Table 4. Sensitivity analysis for the relationship between retirement status and loneliness, excluding those who reported being lonely at wave 4 (short-term) or wave 5 (long- term)

Supplementary Table 5. Sensitivity analysis for the long-term relationship between retirement status and loneliness, by wave

Supplementary Table 6. Sensitivity analysis for the relationship between retirement status and social isolation, model excluding marital status

**Supplementary Table 1. Sensitivity analysis for the relationship between retirement status and loneliness, with alternative loneliness definition categorised as a change in loneliness score from wave 4-5 (short-term) and wave 4-8 (long-term)**

|  | Unadjusted OR  (95% CI) | Adjusted OR*  (95% CI) |
| --- | --- | --- |
| **Short-term (wave 4-5)** | | |
| Not retired | 1.00 (ref) | 1.00 (ref) |
| Newly retired | 0.92 (0.74-1.14) | 0.85 (0.68-1.07) |
| **Long-term (wave 5-8)** | | |
| Not retired | 1.00 (ref) | 1.00 (ref) |
| Newly retired | 1.21 (1.00-1.47) | 1.08 (0.88-1.33) |

*Adjusted for: age, sex, education, marital status, self-reported health, physical activity, wealth, religious

**Supplementary Table 2. Sensitivity analysis for the relationship between retirement status and loneliness, excluding those who retired between wave 5 and wave 8**

|  | Unadjusted OR  (95% CI) | Adjusted OR*  (95% CI) |
| --- | --- | --- |
| **Long-term (wave 5-8)** | | |
| Not retired | 1.00 (ref) | 1.00 (ref) |
| Newly retired | 1.21 (0.84-1.73) | 0.91 (0.60-1.36) |

*Adjusted for: age, sex, education, marital status, self-reported health, physical activity, wealth, religious.

**Supplementary Table 3. Sensitivity analysis for the relationship between retirement status and loneliness, excluding those who retired prior to reaching the pension age**

|  | Unadjusted OR  (95% CI) | Adjusted OR*  (95% CI) |
| --- | --- | --- |
| **Short-term (wave 4-5)** | | |
| Not retired | 1.00 (ref) | 1.00 (ref) |
| Newly retired | 0.89 (0.69-1.37) | 0.93 (0.60-1.23) |
| **Long-term (wave 5-8)** | | |
| Not retired | 1.00 (ref) | 1.00 (ref) |
| Newly retired | 1.12 (0.89-1.40) | 1.02 (0.69-1.32) |

*Adjusted for: age, sex, education, marital status, self-reported health, physical activity, wealth, religious. Short-term analysis newly retired n=571. Long-term analysis newly retired n=526.

**Supplementary Table 4. Sensitivity analysis for the relationship between retirement status and loneliness, excluding those who reported being lonely at wave 4 (short-term) or wave 5 (long- term)**

|  | Unadjusted OR  (95% CI) | Adjusted OR*  (95% CI) |
| --- | --- | --- |
| **Short-term (wave 4-5)** | | |
| Not retired | 1.00 (ref) | 1.00 (ref) |
| Newly retired | 0.81 (0.57-1.15) | 0.74 (0.50-1.07) |
| **Long-term (wave 5-8)** | | |
| Not retired | 1.00 (ref) | 1.00 (ref) |
| Newly retired | 1.02 (0.75-1.39) | 0.87 (0.62-1.21) |

*Adjusted for: age, sex, education, marital status, self-reported health, physical activity, wealth, religious attendance

**Supplementary Table 5. Sensitivity analysis for the long-term relationship between retirement status and loneliness, by wave**

|  | **Wave 6** | | **Wave 7** | | **Wave 8** | |
| --- | --- | --- | --- | --- | --- | --- |
|  | Unadjusted OR  (95% CI) | Adjusted OR*  (95% CI) | Unadjusted OR  (95% CI) | Adjusted OR*  (95% CI) | Unadjusted OR  (95% CI) | Adjusted OR*  (95% CI) |
| Not retired | 1.00  (ref) | 1.00  (ref) | 1.00  (ref) | 1.00  (ref) | 1.00  (ref) | 1.00  (ref) |
| Newly retired | 1.01  (0.82-1.24) | 0.94  (0.75-1.18) | 1.11  (0.90-1.37) | 1.09  (0.86-1.37) | 0.98  (0.79-1.21) | 0.92  (0.73-1.16) |

*Adjusted for: age, sex, education, marital status, self-reported health, physical activity, wealth, religious attendance

**Supplementary Table 6. Sensitivity analysis for the relationship between retirement status and social isolation, model excluding marital status**

|  |  |  | | **Social Isolation** | |
| --- | --- | --- | --- | --- | --- |
|  |  |  |  | Unadjusted OR  (95% CI) | Adjusted OR*  (95% CI) |
| **Short-term (wave 4-5)** | | | | | |
| Not retired |  |  |  | 1.00 (ref) | 1.00 (ref) |
| Newly retired |  |  |  | 0.81 (0.64-1.01) | 0.69 (0.52-0.92) |
| **Long-term (wave 5-8)** | | | | | |
| Not retired |  |  |  | 1.00 (ref) | 1.00 (ref) |
| Newly retired |  |  |  | 1.05 (0.80-1.38) | 0.77 (0.61-0.94) |

*Adjusted for: age, sex, education, self-reported health, physical activity, wealth, religious attendance
